# Refined protocol for newly onset identification in non-obese diabetic mice: an animal-friendly, cost-effective, and efficient alternative

**DOI:** 10.1186/s42826-024-00202-w

**Published:** 2024-04-22

**Authors:** Chia-Chi Liao, Chia-Chun Hsieh, Wei-Chung Shia, Min-Yuan Chou, Chuan-Chuan Huang, Jhih-Hong Lin, Shu-Hsien Lee, Hsiang-Hsuan Sung

**Affiliations:** 1 National Laboratory Animal Center, National Applied Research Laboratories, Taipei, Taiwan; 2 Department of Microbiology and Immunology, National Defense Medical Center, Taipei, Taiwan; 3 Molecular Medicine Laboratory, Department of Research, Changhua Christian Hospital, Changhua, Taiwan; 4 Biomedical Technology and Device Research Lab, Industrial Technology Research Institute, Hsinchu, Taiwan; 5 Graduate Institute of Immunology, National Taiwan University College of Medicine, Taipei, Taiwan; 6 National Laboratory Animal Center, National Applied Research Laboratories, Tainan, Taiwan

**Keywords:** Spontaneous diabetes, Autoimmune diabetes, NOD mice, Ultrasensitive glucose test, Glycosuria, Urine glucose, Glycemia, Blood glucose, Early onset diagnosis

## Abstract

**Background:**

Therapeutic interventions for diabetes are most effective when administered in the newly onset phase, yet determining the exact onset moment can be elusive in practice. Spontaneous autoimmune diabetes among NOD mice appears randomly between 12 and 32 weeks of age with an incidence range from 60 to 90%. Furthermore, the disease often progresses rapidly to severe diabetes within days, resulting in a very short window of newly onset phase, that poses significant challenge in early diagnosis. Conventionally, extensive blood glucose (BG) testing is typically required on large cohorts throughout several months to conduct prospective survey. We incorporated ultrasensitive urine glucose (UG) testing into an ordinary BG survey process, initially aiming to elucidate the lag period required for excessive glucose leaking from blood to urine during diabetes progression in the mouse model.

**Results:**

The observations unexpectedly revealed that small amounts of glucose detected in the urine often coincide with, sometimes even a couple days prior than elevated BG is diagnosed. Accordingly, we conducted the UG-based survey protocol in another cohort that was validated to accurately identified every individual near onset, who could then be confirmed by following few BG tests to fulfill the consecutive BG + criteria. This approach required fewer than 95 BG tests, compared to over 700 tests with traditional BG survey, to diagnose all the 37–38 diabetic mice out of total 60. The average BG level at diagnosis was slightly below 350 mg/dl, lower than the approximately 400 mg/dl observed with conventional BG monitoring.

**Conclusions:**

We demonstrated a near perfect correlation between BG + and ultrasensitive UG + results in prospective survey with no lag period detected under twice weekly of testing frequency. This led to the refined protocol based on surveying with noninvasive UG testing, allowing for the early identification of newly onset diabetic mice with only a few BG tests required per mouse. This protocol significantly reduces the need for extensive blood sampling, lancet usage, labor, and animal distress, aligning with the 3Rs principle. It presents a convenient, accurate, and animal-friendly alternative for early diabetes diagnosis, facilitating research on diagnosis, pathogenesis, prevention, and treatment.

**Supplementary Information:**

The online version contains supplementary material available at 10.1186/s42826-024-00202-w.

## Background

The non-obese diabetic (NOD) mouse is the primary animal model for investigating diabetes resulting from immune dysregulation. Spontaneous autoimmunity begins at 2–3 weeks of age, progressively targeting and destroying the exclusive insulin-producing cells, β cells, in the pancreatic islets of Langerhans. This process ultimately leads to insulin deficiency and the onset of diabetes symptoms resembling human type 1 diabetes between 12 and 32 weeks of age [1, 2]. However, unlike human cases, NOD mouse model exhibits strong sex bias with a higher incidence in female mice ranging from 60 to 90% and a lower incidence in male mice ranging from 20 to 50%, depending on the individual colony [3, 4]. Given the importance of early intervention in autoimmune diseases, identifying mice near or newly diabetes onset is crucial for therapeutic treatments [5–7]. BG testing is the conventional standard for diagnosing diabetes, with a BG level exceeding the threshold (ranging from 200 to 400 mg/dl) in one or two consecutive tests to confirm the overt diabetes state [8–10]. Consequently, extensive BG testing on large cohort spanning the entire onset window, approximately a 5-month period, is typically required to identify these randomly appearing onset mice. Furthermore, the disease often progresses rapidly to become severe diabetes in few days, leaving a very short window of newly onset stage for diagnosis, which makes the identification of newly onset mice very challenging.

The normal state of urine is typically devoid of glucose due to the renal reabsorption process [11], with the maximal reabsorption rate of glucose in the blood estimated at approximately 180 mg/dl [12]. In individuals with diabetes, where BG levels are frequently exceeding, excessive amount of glucose is excreted into the urine. This can be easily detected by color-graded, semi-quantitative, UG strips, providing an alternative and noninvasive diagnostic indicator for diabetes [13]. Conventionally, the sensitivity of UG strips is around 50 mg/dl, and their positive threshold appears to align with BG levels in diabetic conditions, approximately ranging from 180 to 250 mg/dl [14]. Therefore, it is generally believed that glucose detected in urine at such high levels is considered as a lagging indicator that may not be suitable for newly onset diagnosis alone. Some studies have utilized both BG and UG testing together [15–17] but to our knowledge, none of the literature has specifically compared the screening results between BG testing and UG testing in identifying individuals near diabetes onset.

This study was originally aimed to understand the gap window of glucose leaking from blood to urine during the diabetogenic process of NOD mice. Part I study introduced UG testing into a conventional BG-based survey protocol, focusing on systematically observing each diagnostic moment of every individual mouse. Additionally, observation was conducted using ultrasensitive glucose strips, which are 10 times more sensitive than traditional ones, aiming to detect glucose leakage as early as possible. Part I results revealed a highly synchronized correlation between UG + and BG + results when incorporating ultrasensitive UG testing. These observations also suggest that performing BG testing only when the first UG + result is detected in a given mouse can also efficiently identify the day of diabetes onset, similar to surveys conducted solely by regular BG testing. We applied this conclusion to survey another cohort of NOD mouse in Part II study, initially using UG testing alone, followed by validation of onset through subsequent BG testing. This approach resulted in even earlier diagnosis with fewer than 95 BG tests, compared to over 700 BG tests conducted in Part I among 60 mice each. Here, we systematically present the BG levels at each diagnosed moment from both protocols and proposed the utility of ultrasensitive UG testing to better identify newly onset NOD mice, aligning with the 3Rs principle as a refined protocol in animal research.

## Methods

### Mice

All animal experiments in this study were conducted in accordance with the guidelines of the Institutional Animal Care and Use Committee (IACUC) of National Laboratory Animal Center (NLAC), and the study was approved by the IACUC ethics committee under protocol NLAC(TN)-110-M-007-R1 and NLAC-111-M-022-R2. The NOD/ShiLtJ-Narl strain of mice, which originated from NOD/ShiLtJ, were used in this study. They were bred as a substrain for over 25 generations in the AAALAC-accredited facility at NLAC of NARLabs in Taiwan under specific pathogen-free (SPF) conditions. The mice were fed a 5% fat diet (LabDiet 5010 formulation) and provided with water ad libitum. They were housed under a 12-hour light/12-hour dark cycle at room temperature (21 ± 2 °C) with 55–65% relative humidity. Total 120 female mice were used in this study (60 mice each in Part I and Part II experiment), and non-fasting BG and UG measurements were performed between 12 and 32 weeks of age to identify newly onset diabetic individuals for therapeutic antibody treatment [18]. The original estimation was to obtain 72 newly onset mice for 3 predesigned groups, 24 mice in each group of positive control, negative control and experimental group. 12 mice in each group were used to validate the efficacy of treatment and the other half of 12 mice were used to explore the immunological mechanism behind the treatment effect. The incidence was estimated at minimal level of 60% and therefore total 120 mice were subjected for diabetes survey in this report. This study specifically focused on the methodology and data before diabetes onset without concerning the treatment effect. Although a few BG levels might be measured a couple of days after treatment, these few data do not affect the overall conclusions.

### Diabetes monitoring

Non-fasting BG measurements were performed using the ACCU-CHEK Guide glucometer (Roche Diabetes Care GmbH, Germany). Human finger lancets were used without a panhandle to puncture the distal tail vein, taking only 2–3 µl of peripheral blood for each measurement. BG levels above 240 mg/dl were considered as BG+ [13, 19, 20]. The summary of BG data is shown by mean ± SD, and the statistical analysis method is described in the contain. The UG measurement was performed using QUANTOFIX Glucose strips (Item No. 91,348) of MACHEREY-NAGEL, Germany. This test strip is extremely sensitive to detect glucose at 0.005% (w/w, equal to 50 mg/l or 5 mg/dl in this report) concentration which is 10x more sensitive than conventional test strips. Usually, mice naturally urinate during handling, and if not, gentle pressure is applied to the bladder area to promote urination. The last drop on the tip of the urethral meatus, which only contained 2–3 µl urine, was applied to the strip, and then waited for 30 s for the color to stabilize. The ultrasensitive glucose strip results can be visually categorized into seven color grades, ranging from a typical yellow hue to a deep dark green. These colors correspond to glucose concentrations of 0, 5, 10, 25, 50, 100, and 200 mg/dl, respectively. For better contrast to be recognized [21, 22], the fourth level (light green, > 25 mg/dl of glucose) determined by visual evaluation was used as a threshold for binary classification, where UG levels higher than or equal to this color depth were considered positive. In Part I, BG and UG testing were initially conducted together once a week on the same weekday for diabetes survey. In Part II, UG testing were used solely in twice a week frequency to survey positive candidates. BG testing were performed only on UG + individuals starting from the 1st UG + day until cBG + status was observed as diabetes onset.

### Statistical analysis

The statistical methodologies employed in this study included Kaplan-Meier survival analysis for calculating the accumulative incidence of diabetes in animals. For intragroup comparisons within Part I and Part II, blood glucose levels underwent Tukey’s multiple comparison test of ANOVA analysis at a significance level (α) of 0.05. Intergroup comparisons of blood glucose values were performed using unpaired *t* tests, with corresponding *p* values < 0.05 as significant difference in the Results. These statistical approaches facilitated a comprehensive and rigorous examination of diabetes onset and blood glucose dynamics at each diagnosis moment.

## Results

The Part I experiment employed the conventional BG-based survey approach and incorporated ultrasensitive UG testing to investigate their correlation during the development of diabetes. In the Part II experiment, ultrasensitive UG testing were utilized alone to screen positive candidates, who were subjected to intensive BG testing to confirm overt diabetes onset.

### Part I: the appearance of BG + and UG + before diabetes onset

**Fig. 1:**
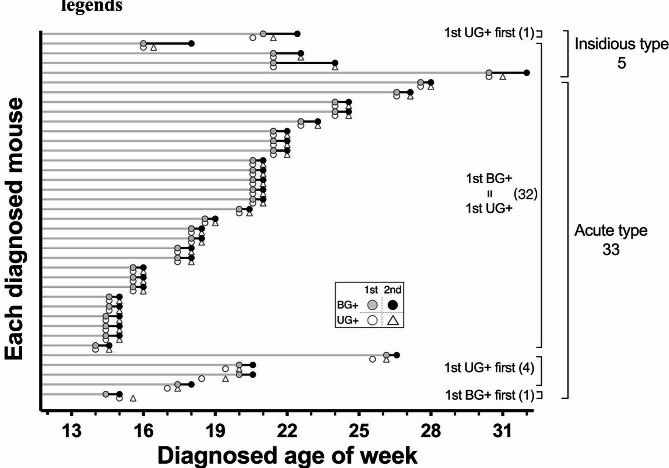
The appearance of BG + and UG + are highly synchronized in Part I during the weekly diabetes survey. Each line represents an individual of diabetic mouse in Part I marked by the age diagnosed for the 1st and 2nd BG+ (gray circle and black circle) alongside the 1st and 2nd UG+ (open circle and open triangle). The period before the diagnosis of 1st BG + is depicted by gray bars, encompassed 263 BG tests in these 38 diabetic mice alone. Additionally, the rest 22 non-diabetic mice underwent 398 BG tests throughout the entire observation window without yielding positive results. The duration between 1st and 2nd BG + are illustrated by black bars. Mice are categorized based on two types of diabetes onset: the acute type (33/38, 86.8%) where 1st and 2nd BG + occur consecutively with short black bars, and the insidious type (5/38, 13.2%), where 2nd BG + is observed a week or longer after 1st BG+, resulting in extended black bars in the upper part of the figure. Most importantly, 32 out of 38 mice (84.2%) exhibit 1st UG + and 1st BG + simultaneously (indicated by vertically overlapping open and gray circles in the middle part). The 5 mice in the 1st UG + first group can be also diagnosed of their own 1st BG + results in the following tests. These findings strongly suggest that during the progression of spontaneous diabetes, the appearance of 1st UG + results precedes or coincides with the 1st BG+, indicating a potential use of ultrasensitive UG test as a primary survey approach

In Part I, the diagnosis primarily focused on BG testing that initially conducted once a week for diabetes survey in 60 female NOD mice. In addition, UG testing were performed on ultrasensitive glucose strips right before BG testing to explore their correlation. The measurement frequency was specifically increased to twice a week when a given individual was first diagnosed of BG+ (> 240 mg/dl) or UG+ (> 25 mg/dl). To elucidate the status near diabetes onset, we plotted the age at which animals were diagnosed with UG + and BG + for the first and second time in Fig. 1, denoted as 1st UG+, 1st BG+, 2nd UG+, and 2nd BG+, respectively. Within the observation period between 12 and 32 weeks of age, there were total 38 mice characterized as overt diabetes which had been diagnosed with two consecutive BG + results, denoted as cBG+. The overall incidence of the female NOD/ShiLtJNarl substrain mice is 63.3% (38/60) shown as accumulative incidence in supplementary Fig. 1 and all the record is attached as supplementary data of worksheet format. Conclusively, in the initial once weekly sampling frequency, 1st UG + and 1st BG + can be detected at the same time in majority of mice 84.2% (32/38) regardless of onset types. Interestingly, there were 5 mice where 1st UG + can even be detected prior than 1st BG + denoted as “UG + first” in Fig. 1. Nonetheless, 4 of these 5 mice were quickly found to be BG + in the next test, while the remaining mouse was found to be BG + 11 days after 1st UG+. Only one mouse presented with BG + prior to UG+ (denoted as BG + first in Fig. 1), who was first diagnosed with 261 mg/dl as 1st BG + but remained normal in UG result until 4 days later in the next test when both BG + and UG + were observed.

### Part I: the timeline of BG + and UG + near diabetes onset

**Fig. 2:**
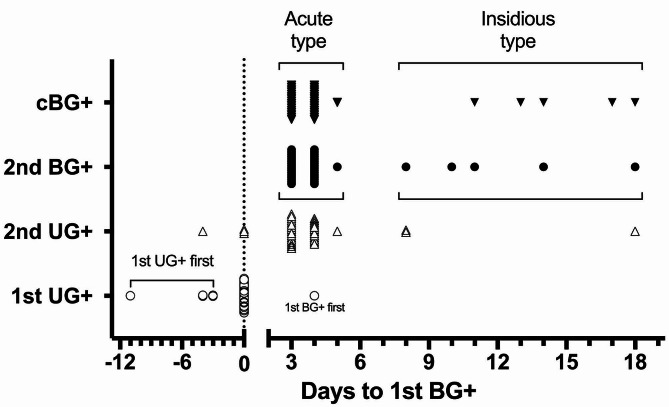
Diabetes onset timeline of Part I. The 1st BG + day of each diabetic mouse in Part I is set as day 0 (aligned by the dashed line) and the corresponding days of diagnosis for 1st UG+, 2nd UG+, 2nd BG+, and cBG + are plotted accordingly to illustrate the onset timeline. The majority of mice (32/38) exhibit a simultaneous diagnosis of both 1st UG + and 1st BG + on the exact same day, represented by condensed open circles on day 0. The 5 open circles on the left indicate their 1st UG + day appearing a couple of days earlier than day 0, forming the 1st UG + first group. In the mouse with 1st BG + first, the 1st UG + day occurs four days after day 0, shown as the only open circle on the right of the dashed line. Symbols in the figure maintain consistency with those in Fig. 1, and group labels are displayed on the y-axis, with additional cBG + denoted by a reversed black triangle. This figure suggests that the UG test can predict the 1st BG + moment effectively and has the potential to replace routine weekly BG survey initially. Furthermore, 2nd BG + and cBG + can be uniformly diagnosed within days 3 to 5 in the acute onset group, a sharp contrast to the insidious onset group. To better distinguish these two onset types, frequent BG tests starting from day 0 are essential. Therefore, we next focus on using ultrasensitive UG tests to identify the 1st BG + moment of individual candidates and subsequently perform intensive BG tests to achieve early diagnosis as soon as possible

To further dissect the near onset window, the day of 1st BG + was set as day 0 and the timeline was analyzed accordingly shown in Fig. 2. Tests were conducted twice a week at that time, so that the output does not reflect daily measurements but provides a general overview of the onset timeline. Majority (33/38) of the 2nd BG + were found right after the 1st BG + within 3 to 4 days in the next scheduled test, fulfilling the definition of cBG + and defined as acute type of onset. However, if the 2nd BG + appeared after day 7, which meant at least one test in between did not meet criteria of BG+, resulting in a prolonged onset duration that classified as the insidious type. Nonetheless, they will still be diagnosed with cBG + within subsequent tests from day 11 to day 18. During this insidious period, the occurrence of 2nd UG + was observed to precede or coincide with 2nd BG + in 3 and 2 cases, respectively (Fig. 1), thereby confirming the indicative role of UG + in predicting the upcoming BG + result. It has also been proposed that the progression of onset possibly reflect the degree of deterioration and potentially affect the outcome of treatment [9, 23, 24]. Therefore, we stratified the data to show these two types of mice accordingly. Most importantly, 1st UG + represented as a leading indicator here that once it is diagnosed, approximately 97.4% of mice (37/38) can be found of 1st BG + within the next 11 days including the majority of 84.2% (32/38) that were found in the same day. This observation also indicated the potential to substitute routine BG testing with UG testing to efficiently address the 1st BG + moment of each NOD mice.

### Part I: the BG testing and level at each moment

**Fig. 3:**
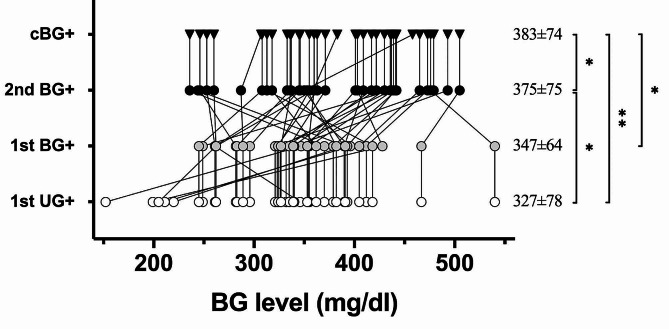
BG levels at each diagnosis moment in Part I. Average BG levels are presented as mean ± SD on the right, analyzed using Tukey’s multiple comparison of ANOVA test. All statistical significances are indicated on the right: one asterisk (*) for *p* value < 0.05 and two asterisks (**) for *p* value < 0.01. Symbols remain consistent with previous figures, and group labels are displayed on the y-axis. In instances where the 1st UG + and 1st BG + are identified on the same day (similarly for 2nd BG + and cBG+), they share the same BG level, represented as vertical lines. The average BG level at the 1st UG + moment is found to be comparable to that at the 1st BG + moment but significantly lower than the levels at the 2nd BG + and cBG + moments, indicating a gradual increasing trend. The BG level of mice confirmed at the cBG + moment is measured at 383 ± 74 mg/dl, representing a definitive onset of newly diagnosed diabetes

Considering the progression of BG level along with diabetes development, we further plotted the change of BG levels of individual mouse at designated moment in Fig. 3. Despite the short interval of only few days, we observed a trend that the average BG level slightly increased from 1st UG + of 327 ± 78 mg/dl to 1st BG + of 347 ± 64 mg/dl, although it didn’t reach the statistic significance (*p* = 0.63). Tukey’s multiple comparison test of ANOVA analysis revealed significance exist in BG levels of 1st UG + vs. 2nd BG+/cBG+, which further protrude the potentially earlier stage of near onset mice at lower BG level detected from 1st UG + candidates. During the comprehensive weekly survey of spontaneous autoimmune diabetes in NOD mice, we observed the high degree of synchronization between UG + and BG + results and figured the 1st UG + result can effectively reflect the 1st BG + status exceeding the predetermined threshold of 240 mg/dl. These findings suggest the existence of small amount of glucose is observed at approximately the same time, sometimes even earlier, then detectable hyperglycemia via BG testing. Considering the enormously efforts to approach these valuable 1st BG + moment, individual sampling window were presented by horizontal grey lines of 38 diabetic mice shown in Fig. 1, which is a total of 263 BG tests till 1st BG+. Subsequent BG testing until cBG + required another 52 BG tests to achieve. Additionally, there were 22 non-diabetic mice that had been undergone 398 BG tests in the entire exam duration, much greater than needed for diabetic ones, but failed to reach the BG + threshold, neither shown in the figures. Taken together, diabetes monitoring of 60 mice took more than 700 BG tests (263 + 52 + 398) across 20 weeks in order to identify 38 newly onset mice in Part I that, according to our current results, can be potentially achieved by faster, cheaper, and non-invasive ultrasensitive UG testing for surrogate.

### Part II: the BG results after UG + have been validated near diabetes onset

**Fig. 4:**
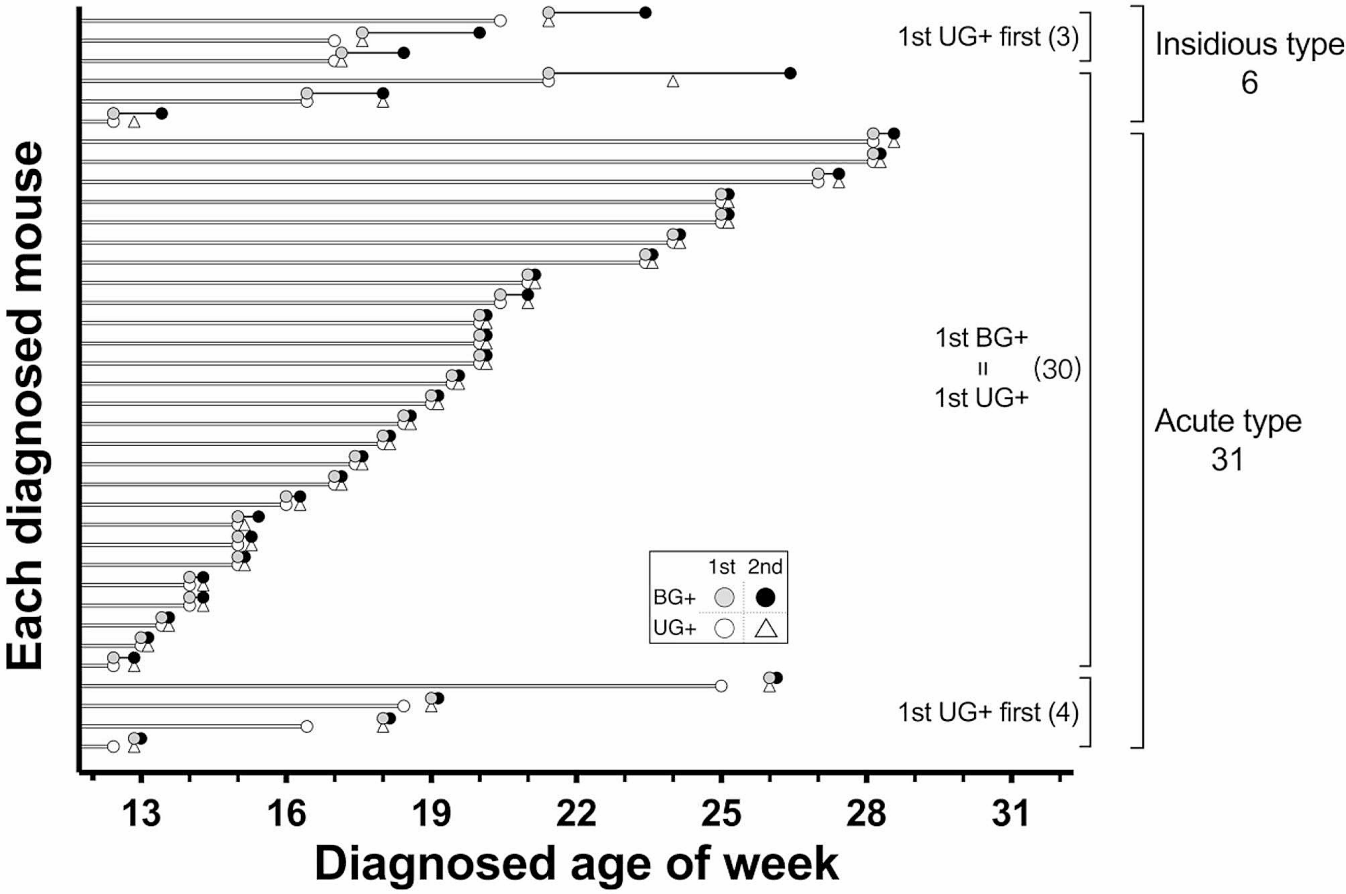
Appearance of UG + and BG + in Part II survey with refined protocol. Each line represents an individual diabetic mouse in Part II. The period surveyed by the UG testing is illustrated by open bars, concluding at the age of each 1st UG + moment represented as open circles. This contrasts with the gray bars of BG testing in Fig. 2, as these have been replaced with the non-invasive UG testing in Part II. BG testing were then immediately performed starting from the day of the 1st UG+, with corresponding ages for the 1st and 2nd BG + marked with gray circles and black circles, respectively. The 2nd UG + is indicated with open triangles, and the duration between 1st and 2nd BG + is represented by black lines. Mice are categorized based on two types of diabetes onset: the acute type (31/37, 83.8%), where 1st and 2nd BG + occur consecutively with very short black lines, and the insidious type (6/37, 16.2%), where the 2nd BG + is observed a week after the 1st BG+, resulting in extended black lines in the upper part of the figure. The majority of mice, 30 out of 37 (81.1%), exhibit their 1st UG + and 1st BG + simultaneously in the same day, indicated by vertically overlapping open and gray circles in the middle part. Using this refined protocol, it takes less than 50 bleedings to identify the 1st BG + moment in these 37 diabetic mice. Additionally, hundreds of negative BG results are saved among the 23 non-diabetic mice. Interestingly, during the twice-weekly UG survey, 7 mice are found to have UG + first (while BG remains normal), followed by their own 1st BG + results in subsequent tests. These findings strongly suggest that during the progression of spontaneous diabetes, the appearance of a small amount of glucose in the urine primarily coincides, and sometimes even precedes, the 1st BG + moment, making the ultrasensitive UG testing an excellent tool to assist in diabetes diagnosis

**Fig. 5:**
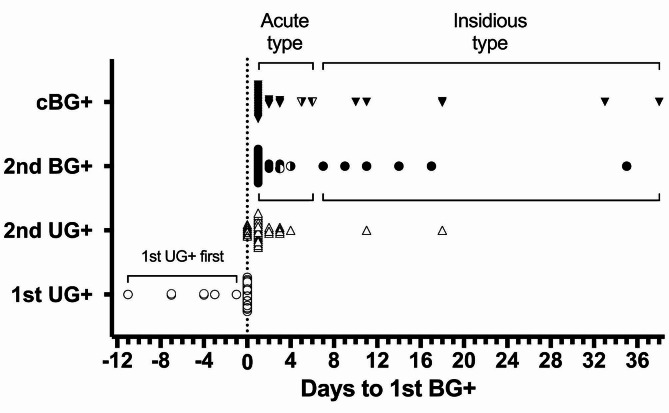
Diabetes onset timeline of Part II. The 1st BG + day of each diabetic mouse in Part II is designated as day 0 (aligned by the dashed line), and the corresponding days of diagnosis for 1st UG+, 2nd UG+, 2nd BG+, and cBG + are plotted to illustrate the onset timeline. The 7 open circles on the left indicate their 1st UG + day occurring 1–11 days earlier than day 0, forming the 1st UG + first group. The remaining mice (30/37) are all diagnosed with their 1st UG + and 1st BG + on the exact same day, represented by condensed open circles on day 0. Symbols in the figure remain consistent with previous figures, and group labels are displayed on the y-axis. Intensive measurements since day 0 reveal that the 2nd BG + can be observed in 22 and 9 mice on day 1 and day 2–4, respectively, comprising all acute types of onset. In contrast, the 2nd BG + appears between day 7 to 35 in the insidious type of onset. We conclude that the 4-day interval from day 0 is crucial for onset determination, especially on day 1 (22/37, 59.5%). When the earliest onset moment is required, daily measurements from day 1 to 4 provide the best resolution (31/37, 83.8%). Moreover, in both types of onset, all the cBG + in both Part I and II can be detected within 4 days from the previous BG+, indicating that the potential fluctuation of BG level (in insidious types of onset) may not exceed 4 days in overt diabetes onset conditions. Considering the frequency of diagnosis substantially determines the definition of “consecutive BG+” with different time intervals, our data suggest that following BG+ (from the previous BG+) within 4 days can be considered as the onset to better identify newly diabetic animals

In Part II, the diagnosis was then focused on UG results to test the detection efficiency. We increased the UG testing frequency to twice a week in another 60 female NOD mice and performed intensive BG testing for 3–4 times a week since the 1st UG + moment. Interestingly, when diagnosed age of UG + and BG + were plotted accordingly as in Fig. 1, identical distribution was observed in Part II shown in Fig. 4. When 1st UG + were detected, 81.1% (30/37) of mice were identified of 1st BG + at the same time. The rest 18.9% (7/37) were first found of 1st UG+, with BG levels ranging from 200 to 234, which appeared approaching but yet reaching the threshold of 240 mg/dl. Nevertheless, 1st BG + can all be subsequently diagnosed within the next few tests after 1st UG + had been detected. Specifically, when the onset timeline is aligned with the 1st BG + day as day 0 in Fig. 5, the 1st UG + result could lead 1 to 11 days ahead of the 1st BG + in this respect. Majority 83.8% (31/37) of the 2nd BG + can be detected within 4 days post 1st BG+, which is nearly the same in Part I of 86.8 (33/38). Considering the normal test results in the previous week, elevated BG exceeding pre-determined threshold (240 mg/dl in this report) can dramatically take place within just few days and these mice are characterized as acute type of diabetes onset (stratified in Figs. 1, 2, 4 and 5). In contrast, there are also minor cases (13.2% in Part I and 16.2% in Part II) characterized as insidious type of onset, where the BG level fluctuated and didn’t get consecutive BG + results until weeks later. These two types of onset can’t be distinguished on the 1st BG + day, which makes the identification of newly onset moment even more challenging and laborious. However, convenient UG survey in Part II allows us to easily focus on intensive BG testing after 1st UG + was detected. The BG sampling interval was reduced to as little as one day, especially within the first few days of diagnosis, compared to the fixed sampling interval of 3–4 days used in Part I. Therefore, 78.4% (29/37) of mice were found of 2nd BG + consecutively on the next scheduled test after 1st BG+ (day 1–3 in Fig. 5) to confirm as newly diabetes onset by only two BG tests each, and in these cases the moment of 2nd BG + and cBG + as well as their corresponding BG levels will be the same. Only two mice showed fluctuation of BG levels transiently below 240 mg/dl between day 1–2 (Fig. 5, half black symbols) that results in delayed of cBG + data to the 3rd BG + moment (consecutive from 2nd BG+) but soon confirmed diagnosis in day 5–6. For cases where 2nd BG + did not appear within 4 days after 1st BG+, we continued the twice-weekly UG survey until next UG + to initiate another round of BG testing.

### Part II: the BG testing and level at each moment

**Fig. 6:**
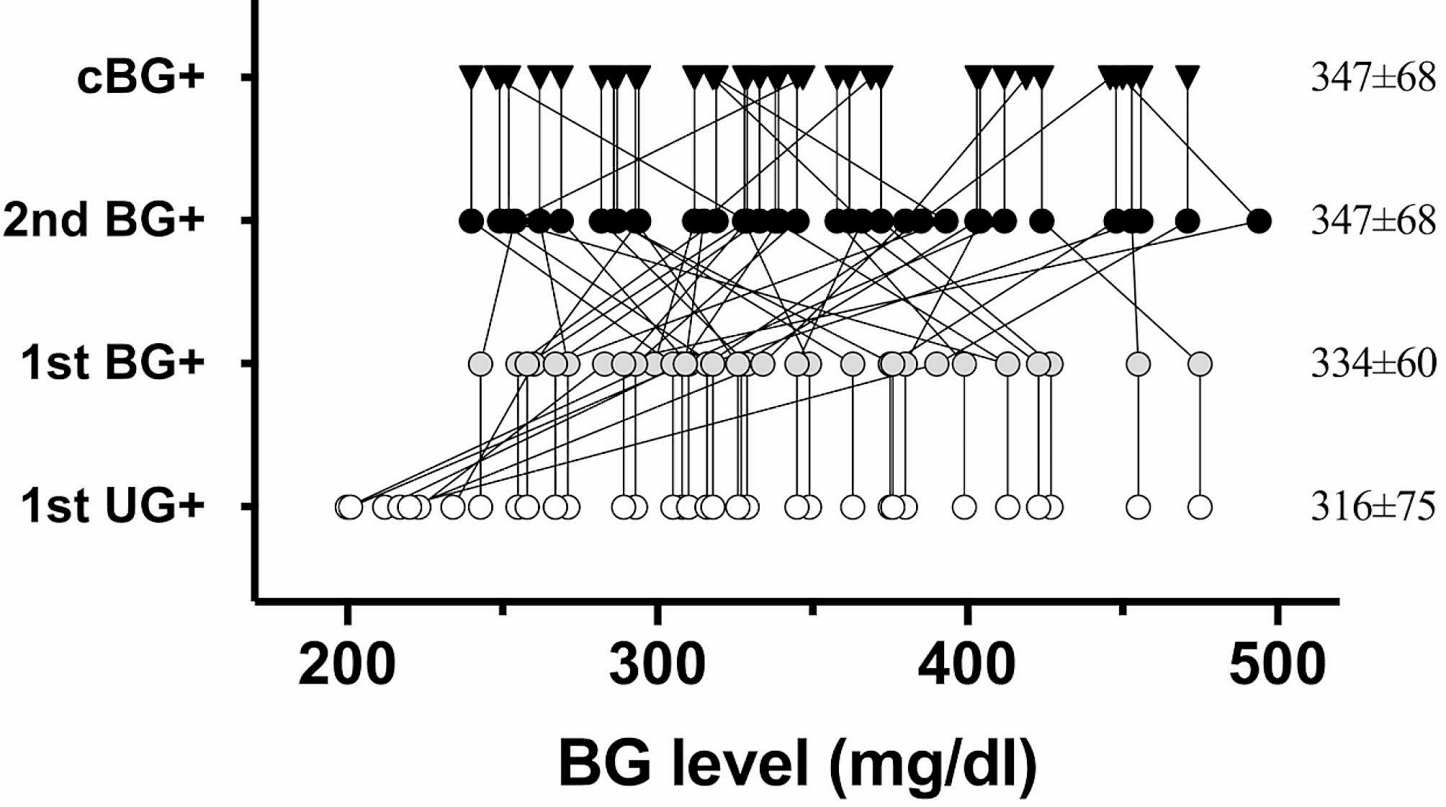
BG levels at each diagnosis moment in Part II. Average BG levels are presented as mean ± SD on the right. Symbols maintain consistency with previous figures, and group labels are displayed on the y-axis. In instances where the 1st UG + and 1st BG + are identified on the same day (similarly for 2nd BG + and cBG+), they share the same BG level, represented as vertical lines. The average BG level at the 1st UG + moment is 316 ± 75 mg/dl, comparable to the cBG + moment of 347 ± 68 mg/dl when overt diabetes is validated. In contrast with the Part I study where cBG level was at 383 ± 74 mg/dl, an unpaired *t* test revealed a significantly lower BG level (*p* = 0.03) screened by this refined protocol. Tukey’s multiple comparison of ANOVA test revealed no statistically significant differences within any two of these values in Part II, suggesting rapid diagnosis within short period (mostly < 4 days) from 1st UG + moment to achieve a more efficient newly onset diagnosis

Although the 7 mice of UG + first group underwent a few additional BG tests, identification of the 1st BG + among total 37 diabetic mice in Part II required 47 BG tests only which in sharp contrast to the 650 BG tests conducted in Part I. Subsequent BG testing until cBG + is validated takes another 46 BG tests. Consequently, diabetes monitoring of 60 mice in Part II took less than 95 BG tests (47 + 46) to identify all the 37 newly onset mice. One of the primary reasons for this efficiency is our refined protocol, which conserved numerous BG tests for the remaining 23 mice (out of a total of 60) that did not develop diabetes (Supplementary Fig. 1). In order to better understand the quality of our diagnosis, the BG level at different moments were plotted in Fig. 6. Average BG levels are comparable at 1st UG + moment between Part I and Part II, measuring 327 ± 78 mg/dl and 316 ± 75 mg/dl, respectively (unpaired *t* test, *p* = 0.536). Nonetheless, intensive BG testing within the initial 4 days since 1st BG + moment substantially reduces the diagnostic period, contributing to significantly lower cBG + level at confirmed diabetes onset between Part I and Part II, measuring 383 ± 74 vs. 347 ± 68 mg/dl (unpaired *t* test, *p* = 0.033). The efficiency of early diagnosis in Part II is further evident in the relatively consistent BG levels from 1st UG + to cBG+, measuring 316 ± 75 to 347 ± 68 mg/dl, with a trend of slight increase but statistically insignificant (Part II, Tukey’s multiple comparison test of ANOVA analysis, α = 0.05). In contrast, twice-weekly BG sampling in Part I (Fig. 3) led to a significant elevation of BG levels from 1st UG + to cBG+, measuring 327 ± 78 to 384 ± 74 mg/dl, emphasizing the importance of intensive BG testing during the initial 4 days following the 1st BG+.

### Summary

Through comprehensive UG testing at ultrasensitive levels performed together with BG testing, we discovered a nearly synchronized correlation between UG levels exceeding 25 mg/dl and elevated non-fasting BG level above 240 mg/dl in NOD mice. As a result, we propose and validate a UG-based survey protocol that proves to be highly useful in diabetes monitoring. There is no difference in terms of onset timing, incidence rate, or BG level at 1st BG + moment surveyed by BG testing in Part I and UG testing in Part II. However, it takes more than 700 BG tests in Part I while UG-based survey of Part II significantly reduced to fewer than 95 BG tests required to complete entire survey of each 60 mice across the 20 weeks onset window. These results suggest that UG surveys can be used as a refinement option to identify near onset mice, but the true timing of onset needs validation by 2nd BG + and/or cBG+, as the disease can fluctuate for a while in small population. The initial 4 days after 1st BG + can be crucial to confirm the onset status. If validation occurs within 4–5 days, it is considered an acute type of onset, which accounts for approximately 85% of the total onset. If validation occurs longer than 7 days, it belongs to insidious type of onset, which accounts for about 15% of the total diabetic mice. Furthermore, the average BG level confirmed at the newly onset moment of cBG + in Part II is below 350 mg/dl, significantly lower than 383 mg/dl in Part I, which also indicates the earlier stage it can detect in the newly onset phase. Since UG survey is noninvasive to animals, more user-friendly, cost-effective, and can shorten the interval time while improving accuracy, we strongly recommend it as a convenient survey protocol for newly onset diabetes in NOD mice.

## Discussion

Diabetes can be monitored by routine BG and UG surveys [25] but continuous BG data are usually preferable over categorical UG data for researchers who initially thought continuous values will be clearer for interpretation. However, BG levels exhibit significant fluctuations near diabetes onset [9, 26] which are often confusing to determining the “consecutive” BG + moment instantly. Extensive BG measurements are therefore required to perform regularly on dozens of mice in order to accurately identify the near onset individuals. While it has been reported that under physiological conditions, the kidney could occasionally excrete small amounts of glucose into urine in cat [27] and dog [28], our own experience with mice indicates that urine is nearly devoid of glucose. The presence of even a small amount of glucose in mice urine suggests that a BG disorder has persisted long enough to surpass the renal reabsorption threshold, becoming evident as an indicator of imminent diabetes onset. Previous studies have reported that glucose is metabolized rapidly in vivo with half-life approximately only 5 minutes [29] and the majority of un-reabsorbed carbohydrate molecules in mouse blood can be excreted into urine within few hours [30]. The short period of excess glucose from blood excreted to urine explains why the majority of the 1st BG + and 1st UG + events (∼ 85%) are co-diagnosed on the same day in diabetic individuals.

Additionally, approximately 15% of animals were detected of UG + prior to their first BG+. These mice cloud be ignored in conventional BG survey as they fail to meet the BG criteria. These UG + first animals must have experienced temporally elevated BG levels then return to normal at the UG + moment. The exact BG level and duration required to cause UG + remains unclear, but transient dysregulation of glucose cloud also indicate the pre-diabetes status typically observed before overt diabetes onset. Our results also suggest that ultrasensitive UG detection is beneficial in identifying these mice, all of which were later confirmed to approach the onset, serving as a reliable indicator for early diagnosis. Only one animal was detected of 1st BG + prior to UG+. At that time, its BG level was slightly higher than the threshold of 240 mg/dl but had not accumulated long enough to trigger UG+. We suspect that this mouse was detected right at the very beginning of BG + but yet accumulate in the urine. Given the once-weekly testing frequency, the probability of such a case is less than 3%, which can be further reduced in the twice-weekly survey process.

In addition to the 38 and 37 animals characterized as diabetic in Part I & II, respectively, three animals were diagnosed with 1st BG+/UG + at different time points, and one animal was UG + but BG-. However, their test results returned to normal throughout the rest of observation window. These findings together with the existence of 15% insidious type of onset mice suggest two positive test results are required to define overt onset. The ability to detect 1st BG + efficiently allows for intensive measurements within the week afterward. Our data suggest that daily measurements within four days after 1st BG + provide the best window of diagnosis, with 84% of 2nd BG + occurring within this time, which covers almost all acute type of mice. However, intensive BG measurement also altered the definition of consecutiveness, which was initially 3–4 days before 1st UG+/BG + then became 1 day afterwards. Once BG level falls below the threshold, two consecutive BG + tests must be re-accumulated to meet the definition of overt onset, as shown in Fig. 5, where two data points of 2nd BG + do not equal with cBG+ (half black symbols). We found that the BG levels of these two animals returned to normal on day 1 but became BG + again on day 3–4. This suggests that the BG levels could potentially fluctuate for 4 days. When the test interval is set to 3–4 days apart as in Part I, it may cross the intermediate fluctuation and directly meet the criteria of cBG+. This also explains why the 2nd BG + and cBG + of the acute type in Part I completely overlap between day 3–5 (Figs. 2 and 5) without any individual of fluctuation. This study focused on efficient diagnosis and we propose the 4 days interval can be crucial in designing the survey protocol. Therefore, we recommend the twice-weekly screening frequency to avoid the detection gap between the potential fluctuations, although once or twice weekly survey did not result in significant difference on 1st BG + level between Part I and II.

Early onset period is crucial for diabetes treatment [7], but screening of NOD mice in the newly onset phase can be laborious and challenging. When BG levels before onset are not absolutely required for studies, conducting massive BG tests to identify near onset mice can be extremely inefficient. Our refined protocol employs a twice-weekly survey to screen UG + candidates then subjected them to intensive BG testing until cBG + results is validated as overt onset. When even more precise onset timing is crucial for certain experiments, we suggest conducting daily BG measurements starting from the day of the 1st BG + for four consecutive days. This will instantly identify acute type of onset and distinguish them from insidious type, which require further testing later. This refined protocol not only improves animal welfare by reducing the stress and discomfort associated with frequent blood sampling but also reduces labor and costs, allowing researchers to diagnose the onset of diabetes more efficiently, cost-effectively, and accurately.

## Conclusions

Our study demonstrated that detecting the small amounts of glucose exceeding approximately 25 mg/dl in the urine serves as a significant indicator for impending diabetes onset in the NOD mouse model. This correlates almost perfectly with BG levels exceeding 240 mg/dl, with no lag period found. Consequently, we have validated the feasibility of applying noninvasive UG testing at the ultrasensitive level to screen UG + candidates for subsequent intensive BG testing, efficiently identifying every newly diabetes onset mouse with few BG tests per mouse. In the cohort of 60 mice, the conventional BG-based survey protocol requires over 700 BG tests, while our UG-based refined protocol takes fewer than 95 BG tests to complete the entire survey. Furthermore, this protocol demonstrates higher sensitivity to detecting newly onset diabetes at lower BG levels (∼ 350 mg/dl) than the conventional approach (∼ 400 mg/dl). Taken together, our study provides an exemplary case as well as the fundamental principle for researchers to better arrange their own survey scheme with minimal effort. Additionally, conducting UG tests is a cost-effective alternative to BG tests, with each UG test costing only approximately 1/10 of a BG test. From the perspective of the 3Rs, this refined protocol potentially saves hundreds of bleeding procedures and unnecessary animal suffering during batch screening, minimizing the associated stress and discomfort and serving as an efficient alternative for the identification of newly onset NOD mice that facilitates research on the diagnosis, pathogenesis, prevention, and treatment of spontaneous autoimmune diabetes.

### Electronic Supplementary Material

Below is the link to the electronic supplementary material.

Supplementary Material 1

## Data Availability

All data are available in the manuscript or upon request to the corresponding author.

## Abbreviations

BG: Blood glucose
NOD mice: Non-obese diabetic mice
UG: Urine glucose
