# Supplementary material for: Refined protocol for newly onset identification in non-obese diabetic mice: an animal-friendly, cost-effective, and efficient alternative
Source: Lab Anim Res. 2024 Apr 22;40:16. doi: 10.1186/s42826-024-00202-w (PMC11034171; doi:10.1186/s42826-024-00202-w)
Supplement: Supplementary file 1 — Supplementary Material 1 [file 42826_2024_202_MOESM1_ESM.docx]

**Additional figure legend**


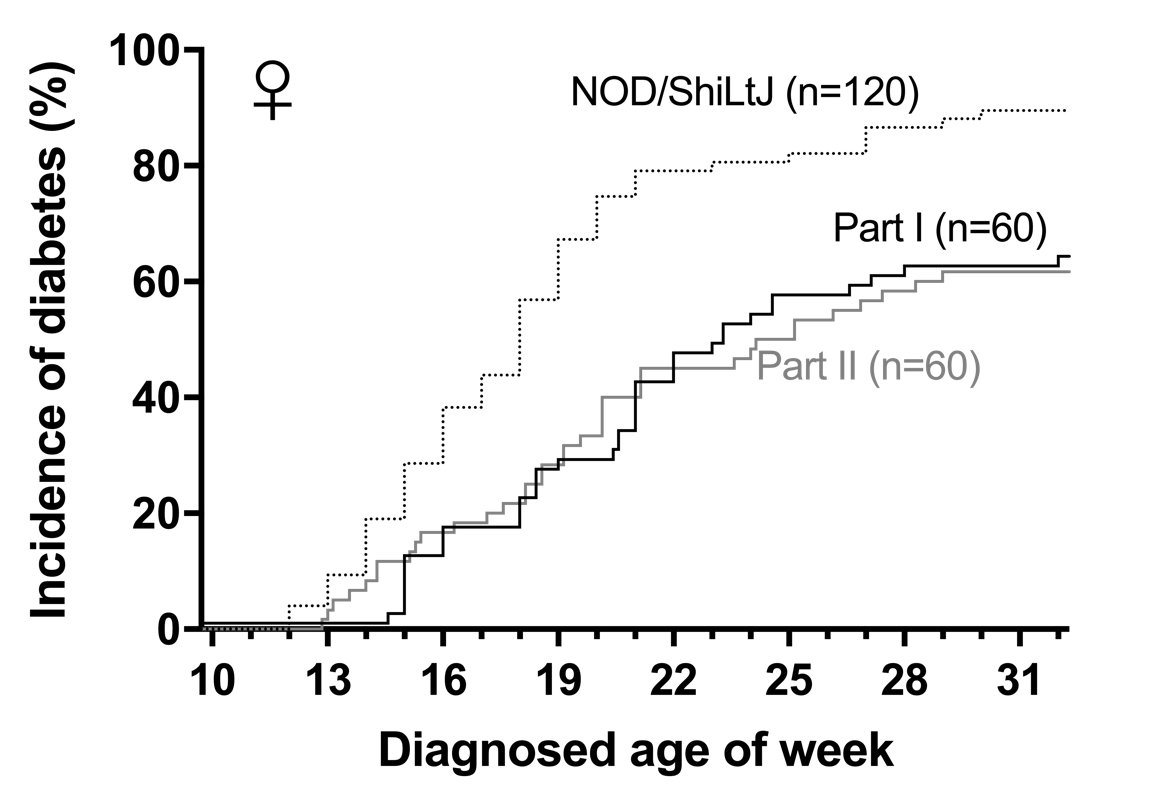


**Supplementary figure 1. Accumulative diabetes incidence of NOD/ShiLtJNarl substrain mice.** Both Part I and II surveyed 60 female mice and the overall incidence is measured as 63.3% (38/60) and 61.2% (37/60), respectively. The curves depict the percentage of mice developing diabetes over time, with Part I employing a once-weekly BG-based survey (solid black line) and Part II utilizing a refined ultrasensitive UG-based survey (solid gray line). Kaplan-Meier survival analysis revealed no significant difference between the onset curves of Part I and Part II (*p >* 0.05), suggesting comparable effectiveness in diabetes survey but different on efforts to identify these newly onset mice (detail described in the contain). Additionally, the accumulative incidence data of the original NOD/ShiLtJ mice, obtained from Jackson Laboratory Stock number [001976](https://www.jax.org/jax-mice-and-services/strain-data-sheet-pages/diabetes-chart-001976) for comparison, showed a significantly higher incidence than both Part I and Part II curves of NOD/ShiLtJNarl substrain mice (*p* < 0.0001, Kaplan-Meier survival analysis).

**Additional file**

Additional file 1: Supplementary data

- Additional file 1
- .xlsx
- Supplementary data
- Record of all UG+/BG+ events with date and BG level
